# Differential in infant, childhood and under-five death clustering among the empowered and non-empowered action group regions in India

**DOI:** 10.1186/s12889-021-11486-1

**Published:** 2021-07-21

**Authors:** Ronak Paul, Rashmi Rashmi, Shobhit Srivastava

**Affiliations:** International Institute for Population Sciences, Mumbai, 400088 India

**Keywords:** Under-five death, Infant death, Early childhood death, Mortality clustering, Death scarring, Action group regions, India

## Abstract

**Background:**

With 8,82,000 deaths in the under-five period, India observed varied intra-state and inter-regional differences across infant and child mortality in 2018. However, scarce literature is present to capture this unusual concentration of mortality in certain families by examining the association of the mortality risks among the siblings of those families along with various unobserved characteristics of the mother. Looking towards the regional and age differential in mortality, this paper attempts to provide evidence for the differential in mortality clustering among infants (aged 0–11 months), children (12–59 months) and under-five (0–59 months) period among mothers from the Empowered Action Group (EAG) and non-EAG regions of India.

**Methods:**

The study used data from the National Family Health Survey (2015–16) which includes all the birth histories of 475,457 women aged 15–49 years. Bivariate and multivariate analyses were used to fulfil the objectives of the study. A two-level random intercept Weibull regression model was used to account for the unexplained mother (family) level heterogeneity.

**Results:**

About 3.3% and 5.9% of infant deaths and 0.8% and 1.6% of childhood deaths were observed in non-EAG and EAG regions respectively. Among them, a higher percentage of infant and child death was observed due to the death of a previous sibling. There were 1.67 times [95% CI: 1.55–1.80] and 1.46 times [CI: 1.37–1.56] higher odds of infant and under-five mortality of index child respectively when the previous sibling at the time of conception of the index child was dead in the non-EAG regions. In contrast, the odds of death scarring (death of previous sibling scars the survival of index child) were 1.38 times [CI: 1.32–1.44] and 1.24 times [CI: 1.20–1.29] higher for infant and under-five mortality respectively in the EAG regions.

**Conclusion:**

The extent of infant and child mortality clustering and unobserved heterogeneity was higher among mothers in the non-EAG regions in comparison to their EAG region counterparts. With the growing situation of under-five mortality clustering in non-EAG states, region-wise interventions are recommended. Additionally, proper care is needed to ameliorate the inter-family variation in mortality risk among the children of both EAG and non-EAG regions throughout their childhood.

## Background

Globally, the rate of infant and child mortality has declined across the countries. However, in India alone, 8,82,000 children under-5 years of age died in 2018 [1]. According to the latest sample registration system data, 32 out of 1000 children died before their first birthday in India in 2018 [2]. Moreover, there are significant intra-state and inter-regional differences in the infant and child mortality rates across India [3]. Several factors appear to be the reason for this inequality in the risk of mortality in Indian children. Mortality clustering among high-risk mothers is a known predictor of the existing inequality in risk of mortality among Indian children [4]. Death clustering refers to the unusual concentration of mortality in certain families, which occurs due to a positive association of the risk of mortality among the siblings of those families [5]. Previous literature from developed and developing countries has shown the increasing trend of death clustering among children [6, 7]. In India, the issue of mortality clustering had also emerged as a serious public health concern [8].

Extant research studies gave evidence of the death clustering phenomenon and talked about factors that explained this phenomenon [4, 9–13]. Mortality clustering occurred among specific families in Brazil and was a result of shared familial characteristics among the siblings of the same family [13]. Unobserved genetic characteristics shared by siblings of the same parent resulted in mortality clustering among Guatemalan children [10]. One study had shown the positive association of maternal education and household wealth status with the mortality clustering in families in a historical Italian population [14]. In India, the socio-economic status of households was positively associated with the risk of infant mortality clustering among specific mothers [4]. Behavioural factors like prenatal care, breastfeeding, and immunization were positively correlated with increased risk of mortality clustering among Indian infants [9]. Another study from India showed that the survival status of the previous child increased the mortality risk of the index child in a family [5]. They termed this phenomenon as “death scarring” where the death of the preceding child scars the survival chances of the index child.

Despite the existing body of literature, the phenomenon of death clustering remains to be explored in its entirety [6]. The prospect of a differential in the occurrence of death clustering across certain population characteristics and/or across vulnerable sub-populations is a potential research area that needs attention. One study from Bangladesh showed that there was a differential in the risk of infant death clustering in areas with and without extensive healthcare services [15]. A rural-urban differential in the risk of infant mortality clustering was also sown in a historical Belgian population [7]. A caste-linked differential in the prospect of infant death clustering was observed among Indian children [16]. A longitudinal study had reviewed the theoretical framework of infant and childhood mortality showing the relevance of all the periods of life on survival [17]. Extant research had shown that mortality over the first 60 months of life is not equally distributed among some families and women [7, 13, 18, 19]. As some families or women do not experience a single under-five death, while few are responsible for most of the deaths. The current paper adds to this small but growing body of literature by providing evidence of differential in the risk of infant, child and under-five mortality clustering across the Empowered Action Group (EAG) and non-EAG regions of India. The Indian states are classified into EAG and non-EAG regions based on key development indicators such that the states belonging to the EAG region significantly lag behind the states in the non-EAG region based on those key human development indicators [20]. The EAG region includes the eight Indian states of Bihar, Chhattisgarh, Jharkhand, Madhya Pradesh, Odisha, Rajasthan, Uttaranchal, and Uttar Pradesh whereas all the other remaining Indian states comprise the non-EAG region. The states in the EAG region have higher infant and child mortality rates compared to the non-EAG states [21, 22]. Owing to such differences, the current study examines the infant, child, and under-five mortality clustering respectively among the Indian mothers in the EAG and non-EAG regions. In this study, infant mortality is termed as deaths occurring before the completion of the first year of life (0–11 months) whereas child mortality includes deaths occurring between 12 to 59 months of a child’s life. Existing studies have shown that while maternal and healthcare-specific factors are responsible for the occurrence of infant deaths, mortality among under-five children occurs due to unfavourable socio-economic attributes in India [23–25]. Thus, it is necessary to examine infant, child and under-five mortality clustering separately [11]. This study hypothesized that there was no differential in infant, child, and under-five death clustering across EAG and non-EAG regions of India. Further, we also hypothesized that there was no association of death scarring with the mortality of Indian children during infancy, childhood, and under-five period across the EAG and non-EAG regions.

## Methods

### Data

This study used the data from the National Family Health Survey (NFHS), which is the Indian version of the Demographic and Health Surveys, conducted during 2015–2016 [26]. To date, four rounds of NFHS have been conducted by the International Institute for Population Sciences (IIPS) in collaboration with the Ministry of Health and Family Welfare (MoHFW), Government of India. The NFHS provides vital information regarding population health, morbidity, insurance coverage, and nutrition for India and each of its 29 states and 7 union territories. We utilized the data on full retrospective birth histories of Indian women (till the date of interview) in the reproductive age group of 15–49 years. The birth history file contains data for 1,315,617 children born from 1970 to 2016 that were collected from 476,619 women. This study uses the mother as a measure of the family interchangeably as data was collected from a single woman of each household. We performed three separate analyses on the complete birth history of mothers. The first analysis examined infant death clustering, the second analysis examined child death clustering whereas the third analysis examined under-five death clustering among the EAG and non-EAG regions separately. Only singleton births were included for analysis. Therefore, the analytical sample size of this study is 1,298,017 children born to 475,457 mothers.

### Statistical methods

We performed bivariate and multivariate analyses to fulfil the objectives of the paper. The bivariate analysis involved examining the distribution of the mothers by frequency of deaths relative to the frequency of births occurring under those mothers. The multivariate analysis involved estimating random intercept survival regression models. The advantage of using survival models is that they curtail the loss of crucial information by taking into account censored observations in the retrospective birth histories [27, 28]. We estimate three sets of survival regression models. In the first set, our event of interest is the survival status of the index child during the infancy period, i.e., within 11 months from birth. All children who died during infancy were coded as “Yes”; otherwise, they were coded as “No”. In the second set, the survival status of the index child during the childhood period, i.e., between 12 to 59 months from birth is the event of interest. We coded those children as “Yes” who died in the childhood period and the rest were coded as “No”. Similarly, under-five mortality, i.e., death within 59 months from birth is the event of interest in the third set of analysis. Those children who died in the under-five period were coded as “Yes” and the rest were coded as “No”. In the survival regression models, we are required to choose the distribution that the time-to-event (survival time) function follows. Based on theoretical knowledge and results documented in existing research we use the Weibull proportional hazards model [11, 13, 27]. The Weibull regression model is appropriate in cases where the hazard of occurrence of a particular event is either monotonically increasing or decreasing. Based on existing knowledge of human mortality, we know that the risk of mortality is highest in the first year of life and decreases simultaneously until 5 years of age [29, 30]. Based on the above-given arguments, our use of Weibull regression models is justified.

In the random intercept Weibull regression models, we included two levels – child (level 1) and mother/family (level 2). The use of a two-level random intercept survival model allows us to take into account unexplained inter-mother (family) variation (heterogeneity) in the risk of mortality in children [31]. We give the Intraclass Correlation Coefficient (ICC) as a measure of mortality clustering of children within the mothers. The ICC at the mother-level is the ratio of variation of the risk of mortality across mothers (2nd level units) to the sum of the variation in the risk of mortality among the children and across their mothers [32]. In multilevel survival models, the ICC is a function of both the individual-level and mother-level variance whose value lies between 0 and 1 [32]. The higher the value of ICC the greater is the risk of mortality clustering among specific mothers. We also give the risk of infant and child mortality in terms of hazard ratios. The hazard ratio for the random intercept survival model gives the risk of infant (or child) mortality for a particular category of an explanatory variable in comparison to the reference category of the explanatory variable given the effect of all other explanatory variables as well as the effect of unobserved factors at the mother-level remain constant [27].

All the above analyses were carried out separately for EAG and non-EAG regions of India to denote differential in death clustering. None of the multivariate models violated the assumption of multicollinearity [33]. All Statistical estimations were performed using the STATA software version 14.2 [34].

### Explanatory variables

Previous studies reveal that the scarring phenomenon plays a major role in mortality clustering among infants and children. Scarring occurs when the death of the previous sibling affects the survival chances of the index child [5, 35]. In our study, we measured scarring by a binary variable that denotes the survival status of the preceding sibling during the time of conception of the index child. If the preceding sibling was alive during the time of conception of the index child, then the records were coded as “Alive” and if the preceding sibling was not alive then they were coded as “Dead” [11, 36]. Taking the survival status of the previous sibling at the time of conception of the index child allows us to understand whether the index child was conceived because of the loss of the preceding child [13].

We also included other child-specific, mother-specific and socio-economic covariates related to infant and child mortality in line with the Mosley-Chen framework of child survival [37, 38]. The child-specific covariates are birth interval preceding the index child (in months), birth order, birth cohort, and gender of the index child (male, female). The mother-specific covariates are mother’s age during birth of index child (in years), anaemia status (not anaemic, moderately anaemic, severely anaemic) and level of education (no formal schooling, up to primary, secondary or higher). The household socio-economic covariates are caste (Other Backward Classes (OBC), Scheduled Castes (SC), Scheduled Tribes (ST) and others), religion (Hinduism, Islam and others), place of residence (rural, urban) and wealth quintile of household (poorest, poorer, middle, richer, richest) respectively. Only those maternal and socio-economic covariates were included assuming that they would be time-invariant over the life course of the mothers.

## Results

### Sample characteristics

As shown in Table 1, 19,222 and 42,457 infant deaths occurred across the non-EAG and EAG regions of India. 13% and 14% of infants in non-EAG and EAG regions respectively, whose previous sibling was not alive at the time of their conception, experienced infant mortality. A higher proportion of dead infants were males in both non-EAG (4%) and EAG (6%) regions. Moreover, infant deaths in both regions were higher among children whose mothers have had no formal schooling and were aged less than 20 years at the time of the birth of their child. Additionally, we find that 4% of rural infants experienced death in the non-EAG region. In the EAG region, this increases to 6% in rural infants. Coming to child mortality, there were 4570 and 11,345 child deaths across the non-EAG and EAG regions of India, as shown in Table 2. Among them, 1.7% and 2.1% of children in non-EAG and EAG regions respectively experienced childhood deaths if their siblings had died by the time of their conception. While 1% of females died between 12 and 59 months after birth in the non-EAG regions, it increases to 2% for female children in the EAG regions. Mothers who never had formal schooling and were aged less than 20 years at childbirth, their children mostly experienced child deaths in both regions. About 1% and 2% of the poorest wealth quintile children experience death in non-EAG and EAG regions respectively.

**Table 1:** Absolute (N) and percentage (%) distribution of births and infant deaths by the child-specific, mother-specific and household socio-economic covariates across the EAG and non-EAG regions of India

Characteristics	Non-EAG Region					EAG Region				
	Births		Infant deaths		Chi-square test for association	Births		Infant deaths		Chi-square test for association
	N	%	N	%		N	%	N	%	
**Survival status of previous sibling at the time of conception of index child**										
Alive	328,133	56.5	8860	2.7	χ2 = 5196.15;*p*-value = 0.001	440,912	61.5	20,085	4.6	χ2 = 6803.33;p-value = 0.001
Dead	16,221	2.8	2114	13.0		38,810	5.4	5495	14.2	
Has no preceding sibling	236,383	40.7	8248	3.5		237,558	33.1	16,877	7.1	
**Birth interval preceding to index child (in months)**										
28 and more months	186,283	32.1	3861	2.1	χ2 = 3212.82;p-value = 0.001	241,742	33.7	7517	3.1	χ2 = 8368.85;p-value = 0.001
19–27 months	102,548	17.7	3257	3.2		154,978	21.6	8684	5.6	
Less than 19 months	55,523	9.6	3856	6.9		83,002	11.6	9379	11.3	
Has no preceding sibling	236,383	40.7	8248	3.5		237,558	33.1	16,877	7.1	
**Birth order of index child**										
1–2	418,270	72.0	13,230	3.2	χ2 = 331.34;p-value = 0.001	433,472	60.4	26,610	6.1	χ2 = 295.55;*p*-value = 0.001
3	90,920	15.7	2914	3.2		130,004	18.1	6526	5.0	
4	40,174	6.9	1516	3.8		75,429	10.5	4202	5.6	
5 and more	31,373	5.4	1562	5.0		78,375	10.9	5119	6.5	
**Birth cohort of index child**										
2010–2016	131,015	22.6	3784	2.9	χ2 = 764.94;p-value = 0.001	170,824	23.8	7978	4.7	χ2 = 2704.81;*p*-value = 0.001
2005–2009	120,026	20.7	3548	3.0		154,081	21.5	8090	5.3	
2000–2004	118,212	20.4	3657	3.1		149,892	20.9	8273	5.5	
1995–1999	103,057	17.7	3394	3.3		124,871	17.4	7913	6.3	
1990–1994	72,790	12.5	2880	4.0		80,846	11.3	6248	7.7	
1970–1989	35,637	6.1	1959	5.5		36,766	5.1	3955	10.8	
**Gender of child**										
Male	306,208	52.7	11,051	3.6	χ2 = 181.01;p-value = 0.001	376,037	52.4	23,506	6.3	χ2 = 156.27;p-value = 0.001
Female	274,529	47.3	8171	3.0		341,243	47.6	18,951	5.6	
**Mother’s age during birth of index child (in years)**										
Less than 20	136,083	23.4	6311	4.6	χ2 = 1037.16;p-value = 0.001	152,112	21.2	13,680	9.0	χ2 = 3396.36;p-value = 0.001
20–24	238,460	41.1	7111	3.0		300,792	41.9	16,219	5.4	
25–29	141,359	24.3	3714	2.6		177,608	24.8	8209	4.6	
30 and more	64,835	11.2	2086	3.2		86,768	12.1	4349	5.0	
**Mother’s anaemia status**										
Not anaemic	297,151	51.2	9165	3.1	χ2 = 137.13;p-value = 0.001	329,252	45.9	18,894	5.7	χ2 = 174.57;*p*-value = 0.001
Moderately anaemic	208,083	35.8	7112	3.4		291,572	40.6	16,957	5.8	
Severely anaemic	75,503	13.0	2945	3.9		96,456	13.4	6606	6.8	
**Mother’s level of education**										
No formal schooling	195,540	33.7	8297	4.2	χ2 = 1148.74;p-value = 0.001	410,678	57.3	27,559	6.7	χ2 = 1429.12;p-value = 0.001
Upto Primary	98,551	17.0	3697	3.8		105,850	14.8	6303	6.0	
Secondary or higher	286,646	49.4	7228	2.5		200,752	28.0	8595	4.3	
**Caste of the household**										
Scheduled Tribes	144,226	24.8	4607	3.2	χ2 = 32.12;p-value = 0.001	101,200	14.1	6092	6.0	χ2 = 369.62;p-value = 0.001
Scheduled Castes	99,908	17.2	3582	3.6		143,735	20.0	9726	6.8	
Other Backward Classes	167,092	28.8	5417	3.2		346,913	48.4	20,337	5.9	
Others	169,511	29.2	5616	3.3		125,432	17.5	6302	5.0	
**Religion of the household**										
Hinduism	346,556	59.7	11,793	3.4	χ2 = 164.16;p-value = 0.001	609,694	85.0	36,773	6.0	χ2 = 99.51;p-value = 0.001
Islam	97,726	16.8	3607	3.7		92,441	12.9	4958	5.4	
Others	136,455	23.5	3822	2.8		15,145	2.1	726	4.8	
**Place of residence**										
Urban	172,151	29.6	4552	2.6	χ2 = 338.86;p-value = 0.001	156,544	21.8	7672	4.9	χ2 = 372.88;p-value = 0.001
Rural	408,586	70.4	14,670	3.6		560,736	78.2	34,785	6.2	
**Household Wealth Quintile**										
Richest	109,350	18.8	2409	2.2	χ2 = 1090.59;p-value = 0.001	78,223	10.9	2765	3.5	χ2 = 1601.63;p-value = 0.001
Richer	130,452	22.5	3636	2.8		93,584	13.0	4518	4.8	
Middle	142,840	24.6	4820	3.4		122,217	17.0	6758	5.5	
Poorer	132,307	22.8	5323	4.0		173,126	24.1	10,908	6.3	
Poorest	65,788	11.3	3034	4.6		250,130	34.9	17,508	7.0	
**Overall**	**580,737**	**100.0**	**19,222**	**3.3**		**717,280**	**100.0**	**42,457**	**5.9**	

Note - χ^2^ shows the value of the chi-square test statistic

**Table 2:** Absolute (N) and percentage (%) distribution of births and child deaths by the child-specific, mother-specific and household socio-economic covariates across the EAG and non-EAG regions of India

Characteristics	Non-EAG Region					EAG Region				
	Births		Child deaths		Chi-square testfor association	Births		Child deaths		Chi-square testfor association
	N	%	N	%		N	%	N	%	
**Survival status of previous sibling at the time of conception of index child**										
Alive	328,133	56.5	2830	0.9	χ2 = 302.62;p-value = 0.001	440,912	61.5	7536	1.7	χ2 = 267.40;p-value = 0.001
Dead	16,221	2.8	283	1.7		38,810	5.4	812	2.1	
Has no preceding sibling	236,383	40.7	1457	0.6		237,558	33.1	2997	1.3	
**Birth interval preceding to index child (in months)**										
28 and more months	186,283	32.1	1185	0.6	χ2 = 624.04;p-value = 0.001	241,742	33.7	2626	1.1	χ2 = 1856.82;p-value = 0.001
19–27 months	102,548	17.7	1081	1.1		154,978	21.6	3237	2.1	
Less than 19 months	55,523	9.6	847	1.5		83,002	11.6	2485	3.0	
Has no preceding sibling	236,383	40.7	1457	0.6		237,558	33.1	2997	1.3	
**Birth order of index child**										
1–2	418,270	72.0	2724	0.7	χ2 = 566.05;p-value = 0.001	433,472	60.4	5891	1.4	χ2 = 399.54;p-value = 0.001
3	90,920	15.7	815	0.9		130,004	18.1	2285	1.8	
4	40,174	6.9	487	1.2		75,429	10.5	1480	2.0	
5 and more	31,373	5.4	544	1.7		78,375	10.9	1689	2.2	
**Birth cohort of index child**										
2010–2016	131,015	22.6	496	0.4	χ2 = 710.11;p-value = 0.001	170,824	23.8	1066	0.6	χ2 = 2404.49;p-value = 0.001
2005–2009	120,026	20.7	806	0.7		154,081	21.5	2104	1.4	
2000–2004	118,212	20.4	963	0.8		149,892	20.9	2376	1.6	
1995–1999	103,057	17.7	950	0.9		124,871	17.4	2520	2.0	
1990–1994	72,790	12.5	782	1.1		80,846	11.3	2056	2.5	
1970–1989	35,637	6.1	573	1.6		36,766	5.1	1223	3.3	
**Gender of child**										
Male	306,208	52.7	2339	0.8	χ2 = 4.42;p-value = 0.036	376,037	52.4	4943	1.3	χ2 = 362.45;p-value = 0.001
Female	274,529	47.3	2231	0.8		341,243	47.6	6402	1.9	
**Mother’s age during birth of index child (in years)**										
Less than 20	136,083	23.4	1382	1.0	χ2 = 126.62;p-value = 0.001	152,112	21.2	2975	2.0	χ2 = 184.82;p-value = 0.001
20–24	238,460	41.1	1681	0.7		300,792	41.9	4373	1.5	
25–29	141,359	24.3	985	0.7		177,608	24.8	2599	1.5	
30 and more	64,835	11.2	522	0.8		86,768	12.1	1398	1.6	
**Mother’s anaemia status**										
Not anaemic	297,151	51.2	2242	0.8	χ2 = 13.57;p-value = 0.001	329,252	45.9	4918	1.5	χ2 = 30.34;*p*-value = 0.001
Moderately anaemic	208,083	35.8	1660	0.8		291,572	40.6	4820	1.7	
Severely anaemic	75,503	13.0	668	0.9		96,456	13.4	1607	1.7	
**Mother’s level of education**										
No formal schooling	195,540	33.7	2404	1.2	χ2 = 973.05;p-value = 0.001	410,678	57.3	8766	2.1	χ2 = 2079.69;p-value = 0.001
Upto Primary	98,551	17.0	920	0.9		105,850	14.8	1347	1.3	
Secondary or higher	286,646	49.4	1246	0.4		200,752	28.0	1232	0.6	
**Caste of the household**										
Scheduled Tribes	144,226	24.8	1576	1.1	χ2 = 251.86;p-value = 0.001	101,200	14.1	2220	2.2	χ2 = 624.39;p-value = 0.001
Scheduled Castes	99,908	17.2	800	0.8		143,735	20.0	2799	1.9	
Other Backward Classes	167,092	28.8	1099	0.7		346,913	48.4	4983	1.4	
Others	169,511	29.2	1095	0.6		125,432	17.5	1343	1.1	
**Religion of the household**										
Hinduism	346,556	59.7	2482	0.7	χ2 = 65.59;p-value = 0.001	609,694	85.0	9716	1.6	χ2 = 9.31;p-value = 0.010
Islam	97,726	16.8	803	0.8		92,441	12.9	1366	1.5	
Others	136,455	23.5	1285	0.9		15,145	2.1	263	1.7	
**Place of residence**										
Urban	172,151	29.6	940	0.5	χ2 = 181.87;p-value = 0.001	156,544	21.8	1717	1.1	χ2 = 302.41;p-value = 0.001
Rural	408,586	70.4	3630	0.9		560,736	78.2	9628	1.7	
**Household Wealth Quintile**										
Richest	109,350	18.8	413	0.4	χ2 = 879.75;p-value = 0.001	78,223	10.9	435	0.6	χ2 = 1584.14;p-value = 0.001
Richer	130,452	22.5	695	0.5		93,584	13.0	911	1.0	
Middle	142,840	24.6	1058	0.7		122,217	17.0	1524	1.2	
Poorer	132,307	22.8	1449	1.1		173,126	24.1	2818	1.6	
Poorest	65,788	11.3	955	1.5		250,130	34.9	5657	2.3	
**Overall**	**580,737**	**100.0**	**4570**	**0.8**		**717,280**	**100.0**	**11,345**	**1.6**	

Note - χ^2^ shows the value of the chi-square test statistic

### Cumulative hazard plot

**Fig. 1:**
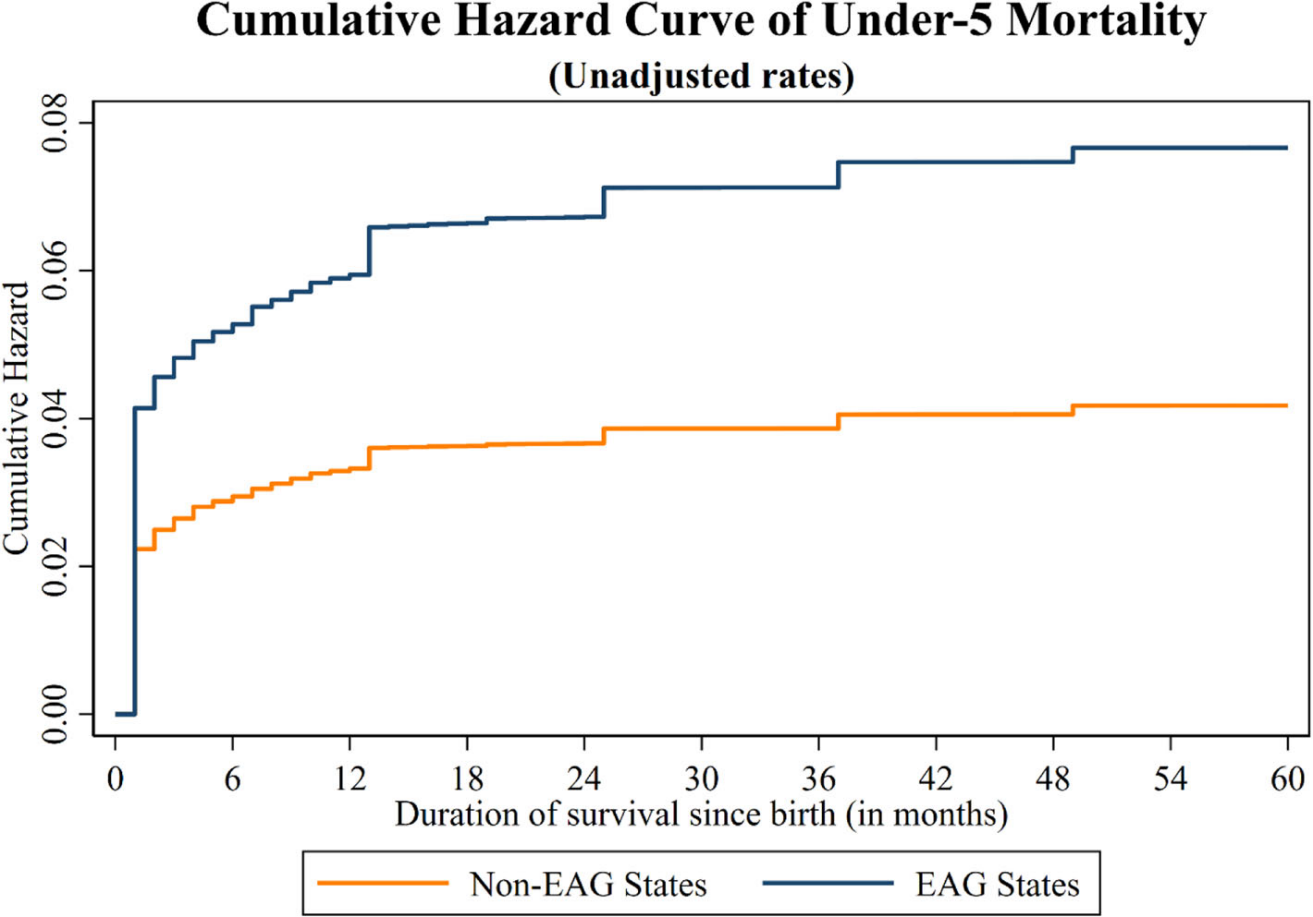
Cumulative Hazard plot for under-five mortality (without adjusting for the effect of explanatory variables) of all children by EAG and non-EAG states in India 2015–16

**Fig. 2:**
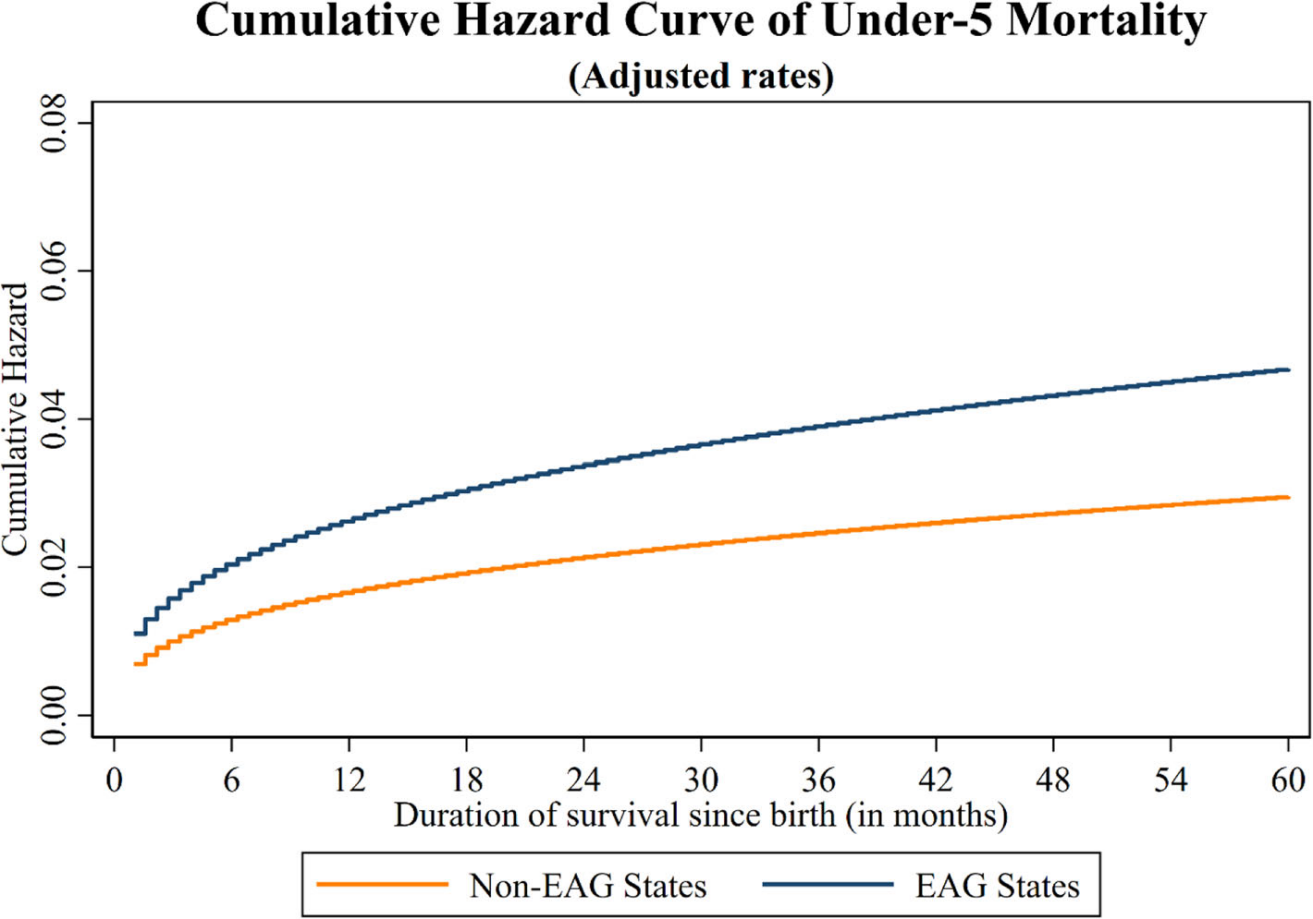
Cumulative Hazard plot for under-five mortality (after adjusting for the effect of explanatory variables) of all children by EAG and non-EAG states in India 2015–16

Figure 1 represents the cumulative hazard plot for under-five mortality (without adjusting for the effect of explanatory variables) of all children by EAG and non-EAG states in India. Moreover, Fig. 2 represents the cumulative hazard plot for under-five mortality (after adjusting for the effect of explanatory variables) of all children by EAG and non-EAG states in India.

### Descriptive analysis

The distribution of births and infant deaths of EAG, non-EAG regions, and India are presented in Table 3. Over 77% of mothers in non-EAG regions and 80% of mothers in EAG regions have two or more births. In the non-EAG and EAG regions, over 15% and 30% of the mothers have five or more children respectively. The result shows an extent clustering of infant deaths within mothers as 7% in non-EAG regions while 15% in EAG regions experienced infant deaths. Table 4 shows the distribution of births and child deaths. The results show that 5% of mothers in EAG regions experience child deaths compared to 2% of mothers from the non-EAG regions. Both tables provide evidence of death clustering within mothers across non-EAG and EAG regions.

**Table 3:** Distribution of women by number of births and infant deaths across the EAG and non-EAG regions of India

	Non-EAG Region		EAG Region		India	
Number of births	Number of women	Percent of women	Number of women	Percent of women	Number of women	Percent of women
1	53,365	22.5	40,288	16.9	93,653	19.7
2	91,058	38.4	65,742	27.6	156,800	33.0
3	51,588	21.8	55,263	23.2	106,851	22.5
4	23,168	9.8	35,861	15.0	59,029	12.4
5	9936	4.2	19,992	8.4	29,928	6.3
6	4422	1.9	10,939	4.6	15,361	3.2
7	1939	0.8	5631	2.4	7570	1.6
8 and more	1593	0.7	4672	2.0	6265	1.3
Total	237,069	100	238,388	100	475,457	100
**Number of infant deaths**						
0	221,022	93.2	204,922	86.0	425,944	89.6
1	13,607	5.7	26,862	11.3	40,469	8.5
2	1922	0.8	4967	2.1	6889	1.4
3	374	0.2	1130	0.5	1504	0.3
4	102	0.0	356	0.1	458	0.1
5 and more	42	0.0	151	0.1	193	0.0
Total	237,069	100	238,388	100	475,457	100

**Table 4:** Distribution of women by number of births and child deaths across the EAG and non-EAG regions of India

	Non-EAG Region		EAG Region		India	
Number of births	Number of women	Percent of women	Number of women	Percent of women	Number of women	Percent of women
1	53,365	22.5	40,288	16.9	93,653	19.7
2	91,058	38.4	65,742	27.6	156,800	33.0
3	51,588	21.8	55,263	23.2	106,851	22.5
4	23,168	9.8	35,861	15.0	59,029	12.4
5	9936	4.2	19,992	8.4	29,928	6.3
6	4422	1.9	10,939	4.6	15,361	3.2
7	1939	0.8	5631	2.4	7570	1.6
8 and more	1593	0.7	4672	2.0	6265	1.3
Total	237,069	100	238,388	100	475,457	100
**Number of child deaths**						
0	232,922	98.3	228,376	95.8	461,298	97.0
1	3798	1.6	8891	3.7	12,689	2.7
2	298	0.1	950	0.4	1248	0.3
3 and more	51	0.0	171	0.1	222	0.0
Total	237,069	100	238,388	100	475,457	100

### The extent of mortality clustering among mothers from non-EAG and EAG regions

Table 5 shows the estimated ICC from multilevel survival analysis for the non-EAG and EAG regions respectively. The mother-level ICC values for null models show that 21% and 14% of the variation in the risk infant mortality in the non-EAG and EAG regions respectively, can be attributed to mother-related characteristics. In the full model, the same values decrease to 14% and 10% for the non-EAG and EAG regions respectively. Overall, we observe that the risk of infant mortality attributable to mothers is higher in the non-EAG region compared to their counterparts in the EAG region. Examining the models for child deaths, the ICC values are markedly higher than the respective models for infant deaths. The full model for child death show mother-level ICC values of 19% and 12% in the non-EAG and EAG regions respectively. The risk of child mortality attributable to mothers is higher in the non-EAG region compared to mothers from the EAG region. Coming to the models for under-5 deaths we observed that 10% (in the non-EAG region) and 6% (EAG region) of variation in the risk of under-5 mortality are attributable to mother-level characteristics in the null model. In the full models, the mother-level ICC values decrease to 7% and 4% for the non-EAG and EAG regions respectively.

**Table 5:** Intercept variance, Intra-class Correlation Coefficient (ICC) and model characteristics from random-intercept Weibull survival regression models of the risk of infant, child and under-five mortality across the EAG and non-EAG regions of India

Infant Death Clustering				
**Measures**	**Non-EAG Region**		**EAG Region**	
	**Null Model**	**Full Model**	**Null Model**	**Full Model**
**Level 2: Mother**				
Variance	1.56	0.96	0.99	0.69
Intraclass Correlation Coefficient (ICC %)	20.91	13.81	14.04	10.25
**Level 1: Children**				
Variance	5.91	5.98	6.04	6.01
Weibull Regression Shape Parameter (γ)***	0.53	0.52	0.52	0.52
Likelihood Ratio Test***	4123.32	940.86	5933.48	1799.09
**No of mothers**	237,069	237,069	238,388	238,388
**No of births**	580,737	580,737	717,280	717,280
**Child Death Clustering**				
**Measures**	**Non-EAG Region**		**EAG Region**	
	**Null Model**	**Full Model**	**Null Model**	**Full Model**
**Level 2: Mother**				
Variance	1.89	1.39	1.21	0.94
Intraclass Correlation Coefficient (ICC %)	24.51	18.81	15.05	11.93
**Level 1: Children**				
Variance	5.81	5.98	6.86	6.94
Weibull Regression Shape Parameter (γ)***	0.53	0.52	0.49	0.49
Likelihood Ratio Test***	834.86	478.38	1296.23	791.28
**No of mothers**	237,069	237,069	238,388	238,388
**No of births**	580,737	580,737	717,280	717,280
**Under-5 Death Clustering**				
**Measures**	**Non-EAG Region**		**EAG Region**	
	**Null Model**	**Full Model**	**Null Model**	**Full Model**
**Level 2: Mother**				
Variance	1.436	0.938	0.837	0.583
Intraclass Correlation Coefficient (ICC %)	9.76	6.53	6.06	4.29
**Level 1: Children**				
Variance	13.278	13.431	12.984	13.017
Weibull Regression Shape Parameter (γ)***	0.35	0.35	0.36	0.36
Likelihood Ratio Test***	4984.38	1339.94	6451.55	2047.43
**No of mothers**	237,069	237,069	238,388	238,388
**No of births**	580,737	580,737	717,280	717,280

Note – (1) Null model is an empty model without any covariates. (2) Full model contains all the covariates. (3) Infant death means death within 0–11 months. (4) Child death means death within 12–59 months. (5) Under-5 death means death within 0–59 months. (6) *** denotes p-value < 0.001

The statistically significant values of the Weibull regression shape parameter (which is less than 1 for all models) point towards a monotonically decreasing risk of mortality during infancy, childhood and under-5 period. This further justifies our choice of the Weibull regression model. Moreover, the statistical significance of the likelihood ratio tests of all models implies that the risk of infant, child and under-5 mortality differs across the mothers.

### Multivariate analysis showing the association of infant, child, and under-5 mortality with relevant explanatory variables in the non-EAG and EAG regions

Table 6 gives hazard ratios showing the association of the risk of infant mortality with the explanatory variables after accounting for mother-level unobserved heterogeneity. In the non-EAG and EAG regions, there are 1.67 times [95% CI:1.55–1.80] and 1.38 times [CI:1.32–1.44] higher risk of infant death when the previous sibling was not alive at the time of conception of the index child. Moreover, the risk of infant death was higher among children born after a birth interval of fewer than 19 months in the non-EAG [OR: 2.71; CI: 2.58–2.84] and EAG [OR: 3.15; CI: 3.05–3.26] regions respectively. Across the non-EAG region, female children have 0.82 times [CI: 0.79–0.84] lower, children whose mother was aged less than 20 years during their birth have 1.32 times [CI: 1.27–1.37] greater risk, and children whose mothers were severely anaemic had 1.20 times [CI:1.15–1.26] higher chances of experiencing infant deaths respectively. Equivalently, across the EAG region, female children have 0.87 times [CI: 0.85–0.89] lower, children whose mother was less than 20 years have 1.37 times [CI: 1.34–1.41] greater risk, and children whose mothers were severely anaemic had 1.20 times [CI: 1.17–1.24] higher chances of experiencing infant deaths respectively. Poorest wealth quintile children were more likely to experience infant mortality in both the non-EAG [OR: 1.73; CI:1.61–1.85] and EAG [OR:1.95; CI:1.85–2.05] regions respectively compared to their counterparts from the richest wealth quintile. Additionally, we observed that rural children were 1.10 times [CI:1.06–1.15] more likely to die during infancy compared to urban children in the non-EAG region.

**Table 6:** Hazard ratios of the risk of infant, child and under-five mortality in the association with relevant child-specific, mother-specific and socio-economic covariates across EAG and non-EAG regions of India using random-intercept Weibull survival regression models respectively

Characteristics	Infant mortality (0–11 months)				Child mortality (12–59 months)				Under-5 mortality (0–59 months)			
	Non-EAG Region		EAG Region		Non-EAG Region		EAG Region		Non-EAG Region		EAG Region	
	HR	95% CI	HR	95% CI	HR	95% CI	HR	95% CI	HR	95% CI	HR	95% CI
**Survival status of previous sibling at the time of conception of index child**												
Alive®												
Dead	1.67*	(1.55–1.80)	1.38*	(1.32–1.44)	1.26*	(1.10–1.43)	0.83*	(0.77–0.90)	1.46*	(1.37–1.56)	1.24*	(1.20–1.29)
Has no preceding sibling	–		–		–		–		–		–	
**Birth interval preceding to index child (in months)**												
28 and more months®												
19–27 months	1.43*	(1.36–1.50)	1.71*	(1.65–1.76)	1.40*	(1.28–1.52)	1.73*	(1.64–1.82)	1.44*	(1.38–1.50)	1.74*	(1.69–1.78)
Less than 19 months	2.71*	(2.58–2.84)	3.15*	(3.05–3.26)	1.86*	(1.70–2.05)	2.43*	(2.29–2.58)	2.55*	(2.44–2.66)	3.05*	(2.97–3.14)
Has no preceding sibling	–		–		–		–		–		–	
**Birth order of index child**												
1–2®												
3	1.11*	(1.06–1.16)	1.04*	(1.00–1.07)	1.20*	(1.10–1.31)	1.17*	(1.11–1.24)	1.12*	(1.07–1.17)	1.07*	(1.04–1.10)
4	1.19*	(1.12–1.26)	1.09*	(1.05–1.14)	1.50*	(1.34–1.68)	1.25*	(1.17–1.34)	1.22*	(1.16–1.29)	1.12*	(1.08–1.16)
5 and more	1.30*	(1.22–1.40)	1.12*	(1.08–1.18)	1.93*	(1.71–2.18)	1.27*	(1.18–1.37)	1.35*	(1.27–1.44)	1.13*	(1.09–1.17)
**Birth cohort of index child**												
2010–2016®												
2005–2009	0.92*	(0.88–0.97)	1.01	(0.98–1.05)	1.25*	(1.11–1.40)	1.53*	(1.42–1.64)	0.83*	(0.80–0.87)	0.94*	(0.91–0.96)
2000–2004	0.94*	(0.89–0.98)	1.05*	(1.02–1.09)	1.51*	(1.35–1.68)	1.78*	(1.65–1.92)	0.88*	(0.84–0.91)	1.00	(0.97–1.03)
1995–1999	0.98	(0.93–1.03)	1.21*	(1.17–1.25)	1.73*	(1.54–1.94)	2.36*	(2.19–2.54)	0.93*	(0.89–0.98)	1.19*	(1.15–1.22)
1990–1994	1.13*	(1.07–1.19)	1.41*	(1.36–1.46)	2.06*	(1.82–2.33)	3.14*	(2.90–3.41)	1.08*	(1.03–1.14)	1.43*	(1.38–1.48)
1970–1989	1.37*	(1.28–1.46)	1.72*	(1.64–1.80)	3.03*	(2.64–3.48)	4.13*	(3.76–4.53)	1.37*	(1.29–1.45)	1.77*	(1.70–1.85)
**Gender of child**												
Male®												
Female	0.82*	(0.79–0.84)	0.87*	(0.85–0.89)	1.07*	(1.01–1.13)	1.44*	(1.38–1.49)	0.86*	(0.84–0.88)	0.97*	(0.95–0.99)
**Mother’s age during birth of index child (in years)**												
20–24®												
Less than 20	1.32*	(1.27–1.37)	1.37*	(1.34–1.41)	1.25*	(1.16–1.36)	1.19*	(1.13–1.26)	1.30*	(1.26–1.35)	1.34*	(1.31–1.37)
25–29	0.98	(0.94–1.02)	0.97	(0.95–1.00)	0.93	(0.85–1.01)	0.98	(0.93–1.04)	0.97	(0.94–1.01)	0.98	(0.95–1.00)
30 and more	1.20*	(1.13–1.27)	1.09*	(1.05–1.14)	0.89	(0.79–1.01)	1.06	(0.98–1.15)	1.14*	(1.08–1.20)	1.09*	(1.05–1.13)
**Mother’s anaemia status**												
Not anaemic®												
Moderately anaemic	1.07*	(1.03–1.11)	1.00	(0.98–1.03)	1.05	(0.98–1.12)	1.07*	(1.02–1.12)	1.07*	(1.04–1.10)	1.02	(1.00–1.04)
Severely anaemic	1.20*	(1.15–1.26)	1.20*	(1.17–1.24)	1.15*	(1.05–1.26)	1.11*	(1.04–1.18)	1.20*	(1.15–1.25)	1.19*	(1.16–1.23)
**Mother’s level of education**												
Secondary or Higher®												
Upto Primary	1.24*	(1.18–1.29)	1.20*	(1.15–1.24)	1.38*	(1.25–1.51)	1.37*	(1.26–1.49)	1.25*	(1.20–1.31)	1.20*	(1.16–1.24)
No formal schooling	1.26*	(1.21–1.31)	1.23*	(1.20–1.27)	1.50*	(1.38–1.63)	1.72*	(1.61–1.85)	1.30*	(1.25–1.35)	1.29*	(1.25–1.33)
**Caste of the household**												
Scheduled Tribes®												
Scheduled Castes	1.08*	(1.02–1.14)	1.19*	(1.15–1.23)	0.80*	(0.73–0.89)	0.98	(0.92–1.04)	1.01	(0.97–1.06)	1.14*	(1.10–1.18)
Other Backward Classes	1.06*	(1.00–1.11)	1.10*	(1.06–1.14)	0.79*	(0.71–0.87)	0.80*	(0.75–0.85)	0.99	(0.95–1.04)	1.02	(0.99–1.05)
Others	1.07*	(1.02–1.13)	1.07*	(1.03–1.12)	0.73*	(0.66–0.81)	0.76*	(0.71–0.83)	0.99	(0.95–1.04)	0.99	(0.96–1.03)
**Religion of the household**												
Hinduism®												
Islam	1.03	(0.99–1.08)	0.93*	(0.90–0.96)	1.07	(0.97–1.18)	1.06	(0.99–1.13)	1.04	(1.00–1.09)	0.96*	(0.93–0.99)
Others	0.87*	(0.83–0.92)	0.87*	(0.80–0.94)	1.17*	(1.07–1.27)	1.03	(0.90–1.19)	0.92*	(0.88–0.96)	0.89*	(0.83–0.96)
**Place of residence**												
Urban®												
Rural	1.10*	(1.06–1.15)	0.97	(0.94–1.00)	1.04	(0.96–1.13)	0.94	(0.89–1.01)	1.10*	(1.06–1.14)	0.97*	(0.94–1.00)
**Household Wealth Quintile**												
Richest®												
Richer	1.16*	(1.09–1.22)	1.32*	(1.25–1.39)	1.18*	(1.04–1.34)	1.55*	(1.37–1.74)	1.16*	(1.10–1.22)	1.36*	(1.29–1.42)
Middle	1.33*	(1.26–1.41)	1.50*	(1.42–1.58)	1.45*	(1.28–1.64)	1.88*	(1.67–2.11)	1.36*	(1.29–1.43)	1.56*	(1.49–1.64)
Poorer	1.56*	(1.47–1.65)	1.71*	(1.62–1.80)	1.99*	(1.75–2.27)	2.32*	(2.07–2.60)	1.64*	(1.56–1.74)	1.82*	(1.73–1.90)
Poorest	1.73*	(1.61–1.85)	1.95*	(1.85–2.05)	2.54*	(2.21–2.93)	3.13*	(2.79–3.51)	1.89*	(1.77–2.01)	2.16*	(2.06–2.26)
**Number of mothers**	**237,069**		**238,388**		**237,069**		**238,388**		**237,069**		**238,388**	
**Number of births**	**580,737**		**717,280**		**580,737**		**717,280**		**580,737**		**717,280**	

Note – (1) *HR* Hazard Ratio; *95% CI* 95% Confidence Intervals. (2) Statistical significance is denoted by asterisks where * denotes p-value < 0.05. (3)® denotes the reference category

The association of the risk of child and under-5 mortality were also observed in the EAG and non-EAG regions of India. The risk of under-5 death was higher among children whose previous sibling was not alive at the time of conception of their conception in the non-EAG [OR: 1.46; CI: 1.37–1.56] and EAG [OR: 1.24; CI: 1.20–1.29] regions respectively. Moreover, children in the non-EAG region were 1.26 times [CI: 1.10–1.47] more likely to experience child mortality if their previous sibling was not alive during their conception. A birth interval of fewer than 19 months makes the children 1.86 times [CI: 1.70–2.05] and 2.43 times [CI: 2.29–2.58] more vulnerable to child mortality in the Non-EAG and EAG regions respectively. Equivalently, under-5 mortality was more likely among children with a preceding birth interval of fewer than 19 months in the non-EAG [OR: 2.55; CI: 2.44–2.66] and EAG [OR: 3.05; CI: 2.97–3.14] regions respectively.

## Discussion

We used the NFHS 2015–16 birth history data to examine whether the extent of infant, child and under-five mortality clustering differs among the mothers from the EAG and non-EAG regions respectively. Existing research has highlighted the importance of maternal and child health care behind the declining rate of infant and child mortality. However, the issue of death clustering using a family approach provides an interesting insight in under-developed countries [35].

We observe that the clustering of infant mortality is higher among mothers in the non-EAG region compared to mothers from the EAG region. Similarly, child and under-five mortality clustering are also higher among the non-EAG region mothers in comparison to their EAG region counterparts. Therefore, this study has clearly shown the existing differential in the infant (aged 0–11 months), child (aged 12–59 months) and under-five (0–59 months) mortality clustering between the EAG and non-EAG regions of India. Further, we have found strong evidence of death scarring during infancy and childhood across the non-EAG regions. However, among EAG regions we have found that the scarring effect was most common during infancy than childhood. This might be because the children who have survived their crucial first year of life were biologically stronger and this reduces their mortality risk at later ages. Both EAG and non-EAG regions show higher mortality risk during infant ages, indicating the vulnerability of a child’s life before their first birthday. The EAG states which lag in the demographic transition have the highest burden of infant and child mortality in India [39].

Our findings suggest that an appropriate birth interval between two children plays an important role in child survival. This finding is similar to earlier studies from India which argue that the birth interval in the model specifies the effect of the replacement hypothesis on the scarring factor [5]. Death scarring occurs when the death of the preceding child reduces the birth interval corresponding to the index child. This reduction in birth interval occurs due to the parents’ desire to replace the dead child by conceiving early [40]. Moreover, the death of a child halts the lactation period which in turn restarts the ovulatory cycle in the mother and enables her to conceive early [41]. This reduction in the birth interval increases the mortality risk of the index child and leads to the clustering of mortality.

Higher birth order was significantly associated with infant and child health. It was also previously argued that higher birth order was directly associated with child and infant mortality [42]**.** It was further argued that the firstborn is observed to be advantaged, the middle-born are observed to be disadvantaged, and the results for later-born are mixed [43].

Among the EAG regions, we find evidence that female children experience less mortality risk during infancy than in childhood. Similar findings were observed in the general population [44] and are consistent with the study of Kumar & Sahu [45] where newborn girls are biologically stronger in their initial ages but as time passes they become vulnerable. This usually happens when gender discrimination in certain families makes the adults more attentive towards the health of the male child.

Maternal age at the time of childbirth is also a prominent factor in child survival and this analysis provides evidence. An immature reproductive system of a young mother may lead to complications in pregnancy that can affect the child further in their life. With a delay in the next birth, women get more time physically and psychologically and this helps in better child development. The findings of the present study are paralleled with the previous literature where it was argued that maternal age at childbirth was a prominent factor for children’s health [46].

Our findings also showed a rich-poor gap in both infant and child mortality risk. This might be due to inequality in health care accessibility and affordability. The financial burden among poor people translates to a greater risk of infant and child mortality. These results were also similar to several studies across India [21, 47].

Mother’s suffering from anaemia had a higher hazard ratio for child and infant death. The findings are consistent with the findings of previous studies where it was argued that maternal anaemia increases the risk for low birth weight, small gestational age babies, and eventually leads to their death [48, 49].

Moreover, a considerable association between the mother’s level of education and child mortality risk was seen. A greater chance of survival among children was found when the mothers have a higher level of education [11]. Mothers with no formal schooling are not aware of the health services to be incurred to secure a child’s health, therefore leading to a higher probability of mortality among infants and children in India [46].

The strengths of this study must be highlighted. Previous studies had brought our attention to the positive correlation of sibling deaths in certain families of India as an important determinant of infant and child death [5, 9, 11, 16, 50]. However, as observed from the literature, this paper may be the first to present a differential in mortality clustering across the EAG and non-EAG regions during infancy, childhood and under-five period respectively. Additionally, there is no available literature that has provided evidence of child death clustering in Indian children. One of the key strengths of this study is that this paper provides evidence of both the scarring effect and unobserved maternal-level factors in infant and child mortality risk across EAG and non-EAG regions of India. Scarring effect was found to be most influential across non-EAG regions after controlling inter-family variation. In contrast, among EAG regions, a lesser chance of scarring effect was seen on children and low infant and child mortality was observed in rural areas after controlling the unobserved mother-level heterogeneity. This might be due to the inclusion of only individual and family-level characteristics to control the time-invariant characteristics. Moreover, existing literature has used a cut-off date before the date of the survey to eradicate the effect of recall bias in their study [10, 13]. Nevertheless, the death risk of any younger sibling depends on the characteristics of the older siblings that they in turn share with their younger siblings. The left truncation of data results in the loss of characteristics related to the older siblings and introduces unobserved bias in the study results [5]. The current study does not suffer from this problem as the complete birth histories of women has been used for analysis.

It is seen that among economically backward regions the role of community is crucial. In this study, community-level characteristics were not used due to their time-inconsistent behaviour. The role of the community cannot be denied behind death clustering. This study has helped us to find inter-family variation but there is a need to show the effect of unobserved heterogeneity at the community level on mortality clustering. Therefore, a major limitation of this study is the need for controlling unobserved community-level factors. Additionally, there can be a chance of recall bias as the information was collected about all the births of women. This limitation can be overcome by the left truncation method but it will create further problems in terms of information loss.

## Conclusion

Despite the above limitations, this study gives crucial insights regarding the role of death clustering and mother-level heterogeneity in the incidence of infant and child mortality across India. Although the vulnerability among EAG regions of India was indicated in past researches, this study reveals that the scarring effect was more common in families of non-EAG regions after the first year of life. Thus, proper care is needed to acknowledge the inter-family variation in mortality risk among the children of both EAG and non-EAG regions throughout their childhood. Moreover, results confirm that the deaths are clustered more under poor and illiterate women, along with children who have a smaller birth interval after the previous child in the family. Therefore, there is a need to promote different programs to concentrate on the mother’s education and sensitize the society towards child healthcare, age at marriage, and birth spacing that ultimately affects both the mother and the child. The findings of this study are helpful for policymakers to identify and target high-risk mothers with programmatic interventions and revisit the strategy for decreasing infant and child mortality rates across the country.

## Data Availability

The study utilizes secondary sources of data that are freely available in the public domain through https://dhsprogram.com/methodology/survey/survey-display-355.cfm. Those who wish to access the data may register at the above link and thereafter can download the required data free of cost.

## Abbreviations

EAG: Empowered Action Group
NFHS: National Family Health Survey
ICC: Intraclass Correlation Coefficient
HR: Hazard Ratio
CI: Confidence Interval
SC: Scheduled Castes
ST: Scheduled Tribes
OBC: Other Backward Classes
